# A 5-gene classifier from the carcinoma-associated fibroblast transcriptomic profile and clinical outcome in colorectal cancer

**DOI:** 10.18632/oncotarget.2237

**Published:** 2014-07-23

**Authors:** Mireia Berdiel-Acer, Antoni Berenguer, Rebeca Sanz-Pamplona, Daniel Cuadras, Xavier Sanjuan, Maria José Paules, Cristina Santos, Ramon Salazar, Victor Moreno, Gabriel Capella, Alberto Villanueva, David G. Molleví

**Affiliations:** ^1^ Translational Research Laboratory, Catalan Institute of Oncology, IDIBELL, L'Hospitalet de Llobregat, Catalonia, Spain; ^2^ Biomarkers and Susceptibility Unit, Cancer Prevention and Monitoring Programme, Catalan Institute of Oncology, IDIBELL, L'Hospitalet de Llobregat, Catalonia, Spain; ^3^ Pathology Department, Hospital Universitari de Bellvitge, IDIBELL, L'Hospitalet de Llobregat, Catalonia, Spain; ^4^ Medical Oncology Department, Catalan Institute of Oncology, IDIBELL, L'Hospitalet de Llobregat, Catalonia, Spain; ^5^ Hereditary Colorectal Cancer Programme, Catalan Institute of Oncology, IDIBELL, L'Hospitalet de Llobregat, Catalonia, Spain

**Keywords:** colorectal, microenvironment, carcinoma-associated fibroblast, prognosis, genetic classifier

## Abstract

Based on 108 differentially expressed genes between carcinoma-associated fibroblasts (CAFs) and paired normal colonic fibroblasts we recently reported, a 5-gene classifier for relapse prediction in Stage II/III colorectal cancer (CRC) was developed. Its predictive value was validated in datasets GSE17538, GSE33113 and GSE14095. An additional validation was performed in a metacohort (n=317) and 142 CRC patients by means of RT-PCR. The 5-gene classifier was significantly associated with increased relapse risk and death from CRC across all validation series of Stage II/III patients used. Multivariate Cox regression analyses confirmed the independent prognostic value of the stromal classifier (HR=2.67; P=0.002). Post-test probabilities provided evidence of the suitability of the 5-gene classifier in clinical practice, identifying a subgroup of Stage-II patients who were at high risk of relapse. Moreover, the a *priory* worst prognosis mesenchymal subtype of tumours can be stratified according to the physiological status of their carcinoma-associated fibroblasts. In conclusion the CAFs-derived 5-gene classifier provides more accurate information about outcome than conventional clinicopathological criteria and it could be useful to take clinical decisions, especially in Stage II. Additionally, the classifier put into relevance the CAF's intratumoral heterogeneity and might contribute to find relevant targets for depleting adequate CAFS subtypes.

## INTRODUCTION

Colorectal cancer (CRC) is one of the most common cancer types in men and women worldwide, with more than one million new cases recorded annually [1]. The difference in prognosis between early and late stages can vary from a five-year survival rate of 93% at Stage-I, to 8% at Stage-IV [2]. For Stage-III patients, post-surgical adjuvant chemotherapy is the standard of care [3]. Although most Stage-II patients are cured by surgery alone, a proportion of them are at high risk of relapse, as determined by clinical and pathological evaluation (advanced T stage, few examined lymph nodes, tumor perforation and low tumor differentiation) and are candidates for adjuvant chemotherapeutic treatment [4]. However, some patients whose clinical factors suggest that they do not have a higher risk of relapse based on clinical factors still relapse, while, on the other hand, approximately 40% of patients with Stage-III CRC enrolled in surgery-only groups did not recur in five years even without adjuvant treatment [5]. These facts demonstrate that the traditional staging system is not sufficient to identify those patients with Stage-II CRC who carry a high risk of poor outcomes, and this may lead to potential under- or over-treatment in many situations. Identification of new biomarkers to improve prediction of high-risk patients with Stage-II CRC and consequently improved individualized cancer care are needed [6].

Many studies have used gene expression profiling to predict the risk of poor outcome in breast [7], lung [8, 9] and prostate [10] cancers, and many other tumor types. In CRC, several studies have developed gene-expression prognostic signatures to classify patients according to their relapse risk [11]. The Oncotype-DX test [12] and Coloprint [13, 14] are now available for Stage-II CRC patients. More recently, an effort to molecularly characterize colorectal cancer has been made [15, 16], emphasizing the high degree of inter-tumor heterogeneity of this entity [17, 18]. Adding further to its complexity, it features intra-tumor heterogeneity, whereby the balance between tumor and stroma and their respective transcriptional status play a crucial role. In that sense, the response of fibroblasts to TGFβ is a mainstream factor for the development of more aggressive tumor cells [19].

Given the importance of stromal elements in modulating and driving cancer progression, it is crucial to determine the contribution of the stroma and to understand the consequences of changes in this tissue compartment. It has been suggested that changes in the tumor microenvironment may benefit the tumor by enhancing proliferation, inducing a more invasive malignant phenotype, and increasing chemoresistance [20, 21]. These stromal changes consist of activation of resident fibroblasts in carcinoma-associated fibroblasts (CAFs), increased matrix deposition, new blood vessel formation and immune cell infiltration. CAFs are the main constituents of tumor stroma and they exist in close proximity to the cancer epithelium. In a previous study (Molecular Oncology 10.1016/j.molonc.2014.04.006), we defined a signature of 108 genes that are deregulated in CAFs from primary tumors relative to normal colonic fibroblasts (NCFs) from adjacent mucosa in CRC patients. The aim of the present work is the development of a stromal genetic classifier from the 108 deregulated genes previously reported by our group. Thus, we defined a 5-gene stromal classifier that could predict the risk of relapse in different independent datasets of whole-tumor samples. To extend this work, we wanted to assess the predictive power of these genes, analyzing expression levels by means of quantitative real-time PCR in a set of 142 fresh samples in order to exploit its clinical value in a more amenable and reproducible technique.

## RESULTS

### Identification of the prognostic stromal classifier

In our previous work (Molecular Oncology 10.1016/j.molonc.2014.04.006), we obtained a list of 108 deregulated genes (DEG) between NCFs and paired CAFs, fitting the following criteria:

- having at least one associated gene;
- mean log RMA expression >4, to exclude background and low-abundance probes,
- standard deviation log RMA expression >0.1, to exclude low-variability probes;
- q-value <0.05;
- >2-fold change for overexpressed genes and <0.5-fold change for underexpressed genes.

We previously reported the functional relationship of those genes in CAFs biology and the processes in which they are involved (Molecular Oncology 10.1016/j.molonc.2014.04.006).

**Figure 1:**
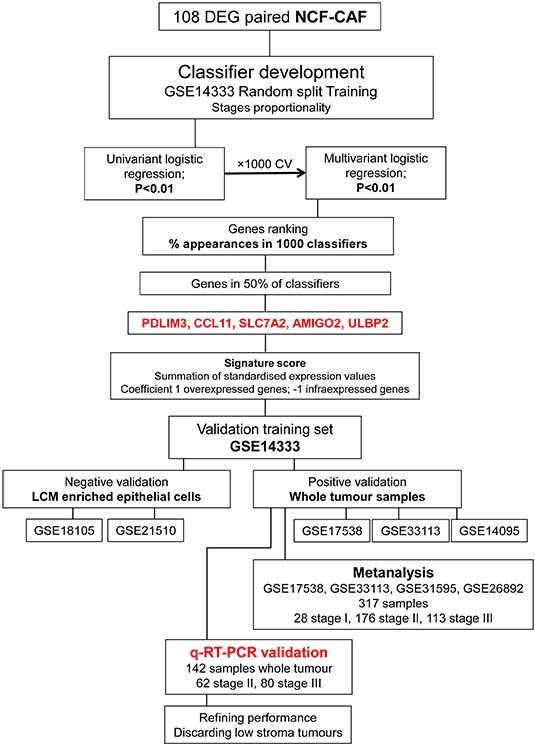
Recurrence classifier development The 5-gene classifier is derived from a 108-gene signature of differentially expressed genes (DEGs) between carcinoma-associated fibroblasts (CAFs) and paired normal colonic fibroblasts (NCFs) (Molecular Oncology 10.1016/j.molonc.2014.04.006). To develop a prognostic classifier we obtained RMA expression values of the 108 DEGs from 135 Stage II and III cases (GSE14333; excluding non-recurrent patients with a follow up of < 3 years; 87 without 48 with recurrence), we used the random resampling procedure to maintain stage proportionality, and divided the initial 135 cases into training and test sets (66% and 33% of cases, respectively). The latter was not involved in gene selection in order to avoid model overfitting. Transcript cluster IDs corresponding to the 108 DEGs (Affymetrix GeneChip Human Gene 1.0 ST Array) between NCF and CAF were mapped to probe set IDs in GSE14333 (Affymetrix Human Genome U133A Plus 2.0 Array). We did a univariate binary logistic regression for each gene, using recurrence as the dependent variable. We chose genes for which p < 0.01 to model an L1 penalized GLMNET multivariate logistic regression in the training set. We then ran the model with the validation dataset. We repeated this process 1000 times, obtaining 1000 classifiers. We recorded all 1000 intermediate signatures, considering only genes and discarding β regression coefficients in order to apply the same biological relevance (same weight) to all genes. We ranked the percentage of times each candidate gene appeared in the signatures, selecting those present in > 50% of signatures for the final classifier. The selected genes were *PDLIM3, AMIGO2, SLC7A2, ULBP2* and *CCL11*. A recurrence score for each sample was computed as the sum of the z-scores of each gene. No gene level parameters were estimated in order to assign the same biological relevance to all five genes (as detailed above), and coefficients were established as 1 for overexpressed genes in CAF *vs*. NCF (*PDLIM3, SLC7A2, ULBP2* and *AMIGO2*; risk genes) and -1 for underexpressed genes in CAF *vs*. NCF (*CCL11*, protective gene). The genes were firstly negatively validated in two datasets of epithelial cell-enriched samples to test their stromal specificity. Moreover, the classifier was validated *in silico* in independent datasets of whole-tumor samples and in an independent cohort of 142 cases of Stage II/III colorectal cancers by quantitative real-time PCR. To conduct the Kaplan-Meier analysis, patients were segregated in two risk groups using the cut off obtained in the training dataset (cut off classifier score = 1.1328; third tertile; > 1.1328 high-risk of relapse; < 1.1328, low-risk of relapse). The third tertile is approximately the relapse prevalence in colorectal cancer (approximately 33.3%).

The aim here is to define a CAFs-derived classifier from the 108 DEG. The entire process of development and validation of the classifier is illustrated in Figure 1. The selected genes were *CCL11* (downregulated in CAFs *versus* paired NCF; protective gene) and *PDLIM3, AMIGO2, SLC7A2, ULBP2* (overexpressed in CAFs *versus* paired NCF).

In the training set, the classifier was not statistically associated with stage (P = 0.629), patient age (P = 0.584), gender (P = 0.808), tumor location (P = 0.144) or adjuvant treatment (P = 0.469).

The expression of classifier genes is mainly fibroblast-specific (Supplementary Figure 1A). To demonstrate that these five genes have prognostic value if the stroma is present in a given sample, we checked our signature in two independent datasets of LCM (Laser Capture Microscope) epithelial cell-enriched samples. As shown in Supplementary Figure 1B and C, the classifier has no predictive power over recurrence in GSE18105 (AUC=0.50) and GSE21510 (AUC=0.53), for which both datasets were enriched in epithelial cells. Additionally, although *ULBP2* can be also expressed in epithelial cells, the contribution of this gene to prognosis is negligible, at least in the two datasets of laser capture enriched samples checked (Supplementary figure 1D and E). We also excluded a possible contribution of cells to the epithelial to mesenchymal transition (EMT) process that would account for the expression of classifier genes (Supplementary Figure 1F-G). Considering these results, we concluded that the epithelial cells did not interfere with EMT masking the performance of the classifier.

### The 5-gene classifier identifies patients with colorectal cancer with poor outcome in three independent *in silico* datasets

**Figure 2:**
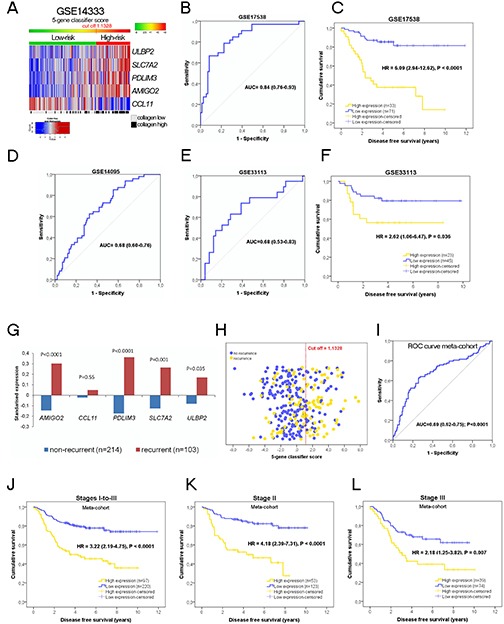
(A) Heatmap of expression values of the 5 genes of the classifier in patients of the training dataset The cut off 1.1328 (the 3^rd^ tertile of the score) segregates patients in two groups of risk. Performance of the 5-gene classifier in the validation dataset GSE17538, **(B)** (ROC curve), and **(C)** Kaplan-Meier analysis of disease free survival. High expression patients (yellow) have a hazard ratio 6.08 times higher to relapse than low expression patients (blue). **(D)** To confirm the prognostic stromal classifier we used another independent dataset (GSE14095), displaying a AUC = 0.68. No survival time information was available for this dataset in order to display Kaplan-Meier curves. An additional validation **(E)** was performed with GSE33113 (Stage II colorectal cancer patients). **(F)** High expression patients (yellow) have a hazard ratio 2.62 times higher to relapse than low expression patients (blue), P = 0.036. In this dataset, for a unit increase in the classifier score, the risk of relapse increases by 1.206 (95% CI = 1.036 - 1.403; P = 0.016). **(G)** Standardized expression values of the five genes in the metanalysis cohort (n=317), including GSE17538, GSE33133, GSE31595 and GSE26892. Four genes (AMIGO2, ULBP2, PDLIM3 and SLC7A2) are significantly higher in recurrent tumors compared to non-recurrent tumors (statistical significance assessed by the Student's t-test). **(H)** Scatter plot of the 317 patients of the meta-cohort according to the 5-gene classifier score (yellow dots recurrent patients, blue dots non-recurrent patients The red dotted line is the cut off obtained in the training dataset to categorize patients according to risk of relapse. The receiver operating characteristic curve describes the performance of the 5-gene classifier in this large cohort **(I)**. **(J to L)** Kaplan-Meier curves in the meta-cohort.

Regarding the cut off obtained in the training dataset (GSE14333) to segregate patients according to relapse risk (3^rd^ tertile, cut off classifier score = 1.1328; Figure 2A), we then chose datasets of whole tumor samples as validation series, thereby ensuring a certain minimum stroma percentage (approximately >20-25%, as detailed at GEO). Consequently, we ascertained the performance of the 5-gene classifier in GSE17538 (Figure 2B and C; AUC=0.84; HR=6.09; P<0.0001), GSE14095 (AUC=0.68; Figure 2D) and GSE33113 (Stage II, Figure 2E and F; AUC=0.68, HR=2.62, P=0.036).

Survival information data were not available for the GSE14095 dataset (recurrence status only).

### *In silico* validation: meta-cohort

We merged the previous GSE17538 and GSE33113 validation datasets, and added two more datasets, GSE31595 and GSE26892, to create a meta-cohort of 317 patients (28 Stage-I, 176 Stage-II and 113 Stage-III). Since the score was calculated using z-scores for each gene and dataset before merging, the impact of the centre/hospital bias was negligible. The association of each single gene of the classifier with the recurrence status is illustrated in Figure 2G and the performance of the classifier in predicting recurrence is depicted in Figures 2H and I. Therefore, according to the cut off (1.1328), 30.6% and 69.4% of patients with the higher 5-gene signature score were identified respectively as high- and low-risk. Low-risk patients had a 5-year DFS rate of 73%, whereas the rate in high-risk patients was only 36% (Figure 2J). Using the 5-gene signature score as a continuous variable, the HR increased by 1.236 (95% CI=1.15-1.33) per unit increase in the SD of expression. Stratifying by disease stage (Figures 2K and L) revealed 5-year DFS rates of 33% in high-risk Stage-II patients, 77% in low-risk patients (HR=4.18, 95% CI=2.39-7.31, P<0.0001) and HR=2.18 (95% CI=1.25-3.82, P =0.007) for high-risk Stage-III patients (5-year DFS rate of 33% versus 59% of low-risk).

To assess the potential utility of the 5-gene classifier in clinical practice we evaluated the post-test recurrence probabilities and compared them with the *a priori* expected prevalence (Table 2).

For this metacohort, a detailed comparison of the 5-gene classifier with clinical factors was not possible because limited information across all GEO datasets further than age, gender and stage. Other variables such as tumor grade was only available for GSE17538 and adjuvant treatment, isolated nodes, lymphatic invasion were not available.

### PCR validation in an independent cohort of 142 patients

We aimed to translate the 5-gene signature score obtained by means of microarray technology to a technique that was more convenient and less subject to bias, such as real-time PCR. For this purpose we used a cohort of 142 well annotated Stage II/III colorectal cancer patients from our institution. Patient characteristics are summarized in Table 1.

**Table 1:** Patient demografics and clinical characteristics for the training set, *in silico* validation metacohort and real timeNAPCR validation set

	Training setGSE14333	Validation *in silico* metacohort	Validation PCR dataset
**Cohort size**	135	317	142
**Gender**			
*Male*	76 (56.3)	104 (51.2)^*^	80 (56.3)
*Female*	59 (43.7)	99 (48.8)^*^	62 (43.7)
**Mean age**	64.38	66.91^*^	66.71
**Stage**			
*Stage I*	NA	28 (8.8)	NA
*Stage II*	64 (47.4)	176 (55.5)	62 (43.7)
*Stage III*	71 (52.6)	113 (35.6)	80 (56.3)
**Location**			
*Colon*	118 (87.4)	NA	91 (64.1)
*Rectum*	17 (12.6)	NA	51 (35.9)
**Grade**			
*Low*	NA	NA	128 (90.1)
*High*	NA	NA	14 (9.9)
**Isolated nodules**			
*>14*	NA	NA	96 (67.6)
*<14*	NA	NA	46 (32.4)
**Adjuvant chemotherapy**			
*yes*	69 (51.1)	NA	74 (52.1)
*no*	66 (48.9)	NA	68 (47.9)
**Recurrence**			
*yes*	48 (35.6)	103 (32.5)	43 (30.3)
*no*	87 (64.4)	214 (67.5)	99 (69.7)
**Kras status**			
*wt*	NA	NA	73 (51.4)
*mut*	NA	NA	47 (33.1)
*NA*	NA	NA	22 (15.5
**MSI status**			
*high*	NA	NA	19 (13.4)
*MSS*	NA	NA	100 (70.4)
*NA*	NA	NA	23 (16.2)

Abbreviations: NA not available or not reported by authors to GEO.

* Available only for 203 patients.% are reported in brackets. Training and validation datasets used were relatively similar in relation to gender, age and recurrence status. Additionally, PCR validation cohort was very similar to the training dataset (GSE14333), with respect to the number of patients, stage distribution and percentage of patients treated in adjuvancy. There were some differences involving the distribution of tumour location in the colon and rectum. A previous report by our group demonstrated that there are no differences at the molecular level between tumours of the colon and rectum [(SanzNAPamplona R, Cordero D, Berenguer A, *et al.* Gene expression differences between colon and rectum tumors. *Clin Cancer Res*;**17**:7303NA12.).

**Figure 3:**
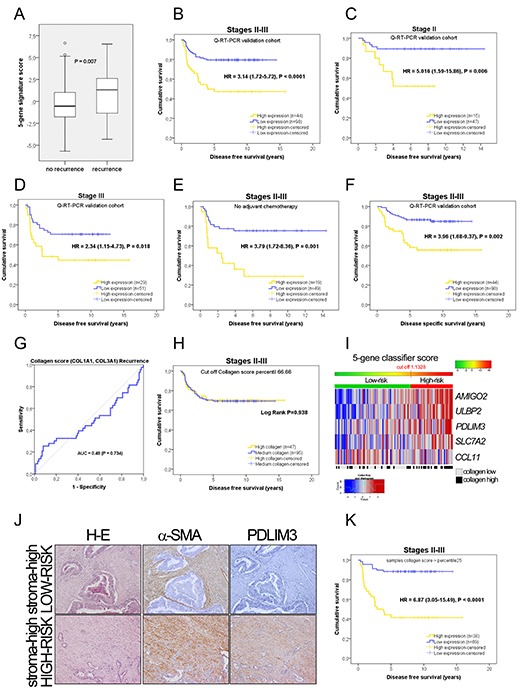
Prognostic information in the PCR independent validation **(A)** Mean values of the 5-gene signature score according to patient recurrence in the PCR cohort of 142 samples (43 recurrent and 99 non-recurrent); t-test, P = 0.007. **(B-D)** Kaplan-Meier plots for disease-free survival in all stages and Stage II or Stage III alone. Considering only the 68 patients who did not receive adjuvant chemotherapy, the 5-gene classifier is associated with recurrence (HR = 3.79, 95% CI = 1.72 – 8.36, P = 0.001; Figure 3E). **(F)** Kaplan-Meier plots for disease-specific survival in all stages. **(G)** Receiver operating characteristic curve describing the absence of predictive power for the recurrence of the collagen score. **(H)** Using the 3^rd^ tertile of the collagen score as a cut off, Kaplan-Meier survival plot shows that the collagen score provides no prognostic information in terms of disease-free survival in the PCR cohort. Thus, tumors with high collagen expression have the same outcome than low collagen tumors. The collagen score does not provide prognostic information. Higher collagen values are not associated with a worse outcome. **(I)** Heatmap showing individual genes included in the classifier on the basis of their expression in the PCR cohort. The cut-off value obtained from the training dataset is represented by the change from green to red in the horizontal bar over the heatmap, which defines the two risk groups. The light grey and black boxes below the heatmap depict the samples identified as low or high collagen according to the collagen score (defined as the average expression of *COL1A*1 and *COL3A1*). Our results suggest that the prognostic value is determined by the physiological status of the CAFs rather than their quantity. In these photomicrographs **(J)** we illustrate two colorectal tumors, both considered as “high stroma” because of the large quantity of desmoplasia (H-E staining), strong positivity for alpha smooth muscle actin (α-SMA), but reflecting a distinct transcriptomic status with respect to PDLIM3 staining. In the top right panel, CAFs display no staining for PDLIM3, one of the five genes of the classifier, and in addition, the tumor is classified as low-risk, according to the mRNA expression values of the five genes. Conversely, in the bottom right panel, CAFs display intense PDLIM3 staining, and the tumor is considered high-risk when considering the expression of all five genes. **(K)** Our hypothesis is that the performance of the 5-gene classifier increases if considering specimens with a minimum number of fibroblasts. Samples with very low levels of fibroblasts will have a very poor representation of mRNA transcripts from fibroblast origin and will therefore be misclassified. As a proof of concept, we selected samples with a collagen score above the 25^th^ percentile, excluding samples with poor collagen scores. This proof of concept can be done since low Collagen score tumors have the same outcome than high Collagen score tumors, as illustrated above. Additionally the proportion of stages and events is maintained in this subanalysis. Kaplan-Meier survival plots after excluding samples below the 25^th^ percentile of the collagen score (n = 107 patients). The 5-gene classifier identifies two risks groups. Five-year DFS and DSS (not shown) survival rates according to the 5-gene signature score improved significantly excluding very low stroma patients.

As depicted in Figure 3A, recurrent samples clearly have higher classifier scores than non-recurrent samples (P=0.007). In this cohort, using the cut off obtained in the training dataset, 31% of patients were considered high-risk (44 high-expression patients, Figure 3B, HR=3.14) whereas 69% were classified as low-risk (98 patients; 48% Stage-II and 52% Stage-III). Fifteen of 62 Stage-II patients were identified as high-risk according to the 5-gene classifier, only two of them being pT4. Low-risk patients had a five-year DFS rate of 80%, while that of high-risk patients was only 45%. Considering the stages separately, Stage-II low- and high-risk patients had five-year DFS rates of 89% and 52%, respectively (Figure 3C). In Stage-III, the five-year DFS rates were 71% and 41% for low- and high-risk patients, respectively (Figure 3D). For DSS, the five-year life expectancy was clearly better for low-risk patients (84% versus 54%, HR=3.96, Figure 3F). High-grade tumors were more often classified as high-risk (P<0.0001) although the outcome may be biased by the high proportion of low-grade tumors (90.1%). High-risk has a statistical association with recurrence, time to recurrence and time to cancer-specific death (Supplementary table 1).

In the univariate analysis the 5-gene classifier was the strongest predictor of DFS (HR=3.14, 95% CI=1.72-5.72, P<0.0001; Table 3). Using the 5-gene signature score as a continuous variable, the HR increased by 1.191 (95% CI=1.05-1.35, P=0.005) per unit increase in the SD of expression. Stage-III, high grade and lymphatic invasion were also associated with poor prognosis in univariate analyses of this cohort. These variables were included in the multivariate analysis (Table 3), in which the 5-gene classifier proved to be the only independent predictor of recurrence (HR=2.67, 95% CI=1.42-5, P=0.002).

Post-test probabilities confirmed the clinical utility of the 5-gene classifier for clinical decision-making, most notably in Stage-II patients (Table 2).

**Table 2:** Post test recurrence probabilities values for the clinical value of the 5-gene signature score in each validation dataset

		Prevalence	LR+	LR−	Positive post test probability	Negative post test probability	Difference
*In silico* Meta-cohort (n=317)	All stages	0.325 (0.27-0.38)	2.61 (1.88-3.6)	0.59 (0.48-0.73)	0.56 (0.45-0.65)	0.22 (0.17-0.28)	0.34
	Stage I	0.11 (0.02-0.28)	2.08 (0.33-13.05)	0.79 (0.35-1.79)	0.2 (0.01-0.7)	0.08 (0.015-0.29)	0.12
	Stage II	0.29 (0.22-0.36)	2.96 (1.92-4.56)	0.53 (0.38-0.73)	0.55 (0.41-0.68)	0.18 (0.12-0.26)	0.37
	Stage III	0.43 (0.34-0.53)	2.09 (1.23-3.53)	0.67 (0.5-0.88)	0.62 (0.45-0.76)	0.34 (0.23-0.45)	0.28
PCR validation Initial cohort (n=142)	All stages	0.30 (0.23-0.39)	2.52 (1.57-4.04)	0.59 (0.42-0.82)	0.52 (0.37-0.67)	0.20 (0.13-0.3)	0.32
	Stage II	0.19 (0.11-0.32)	3.65 (1.64-8.07)	0.50 (0.25-0.97)	0.47 (0.22-0.73)	0.11 (0.04-0.24)	0.36
	Stage III	0.39 (0.28-0.5)	1.95 (1.1-3.46)	0.66 (0.45-0.96)	0.55 (0.36-0.73)	0.29 (0.18-0.44)	0.26
PCR validation Collagen score Percentil25 (n=107)	All stages	0.28 (0.2-0.38)	3.53 (2.17-5.74)	0.34 (0.18-0.61)	0.58 (0.41-0.73)	0.12 (0.05-0.22)	0.46
	Stage II	0.17 (0.08-0.3)	7.17 (3.02-17)	0.14 (0.02-0.89)	0.58 (0.29-0.83)	0.02 (0.001-0.16)	0.56
	Stage III	0.38 (0.26-0.51)	2.23 (1.26-3.94)	0.46 (0.24-0.86)	0.58 (0.35-0.75)	0.22 (0.1-0.4)	0.36

To assess the potential usefulness of the 5-gene signature on clinical practice we evaluate the post test recurrence probabilities and we compared them to the *a priori* expected event proportion. For the *in silico* meta-cohort, considering all stages (expected recurrence value 32.5%) the post-test recurrence probability for the high-risk group increased to 56% and for the low-risk group the prediction was 22% probability of recurrence (34% absolute difference). Stratifying by stage, Stage I patients recurrence prevalence was 11%, but the post test probabilities increases to 20% for high-risk patients and decreases to 8% for low-risk patients. The 5-gene classifier had more potential clinical value for stages II-III. The absolute difference between positive post-test probability and negative post-test probability was 37% for stage II and 28% for stage III patients. On the other hand, for PCR cohort, considering all stages (expected recurrence value 30%) the probability of recurrence bearing a tumour with high expression of the 5-gene classifier increased to 52% and decreased to 20% in low expression tumours. In stage II the expected probability of relapse is 19%. According to the 5-gene classifier, bearing a high expression tumour, this value increases to 47% and the value decreased to 11% in case of low-risk tumours. Thus, the absolute difference is 36%.

From the PCR cohort, considering samples with Collagen score > percentile25, the clinical value of the 5-gene classifier increases and is especially remarkable for stage II patients. As an example, selecting samples with Collagen score above percentile25 (n=107), the *a priori* probability of relapse of a stage II patient is 17%. Applying the 5-gene classifier, the probability of relapse will increase to 58% in case of bearing a high expression tumour, and will decrease to only 2% in case of a low expression tumour.

### Physiological status rather than the quantity of CAFs confers prognostic value

We hypothesized that the prognostic value of the 5-gene classifier reflects the physiological state of the CAFs rather than the number of fibroblasts in the tumor, and that samples with a very low percentage of CAFs might be misclassified since low levels of specific mRNAs would be present in the sample to be amplified among the large quantity of mRNA from other cell types. To test this, we obtained the most fibroblast-specific genes from GSE39396 (Supplementary figure 2A) and selected the two with the least variation in CAFs: *COL1A1*, and *COL3A1*. These genes were also associated specifically with CAFs from the breast [22]. We first demonstrated *in vitro* that there is a correlation between the number of fibroblasts in a sample and the expression of a collagen score (average expression of *COL1A1, COL3A1*) independently of the type of fibroblasts used (Supplementary Figure 2B, top panels). On the other hand, there was no correlation with the five genes of the classifier (Supplementary Figure 2B, bottom panels). Thus, the 5-gene classifier reflects the physiological state of the CAFs rather than the number of fibroblasts in a sample, and different CAF phenotypes are probably present in the same tumor, demonstrating the heterogeneity of the CAFs (Supplementary Figure 2C). In applying this assumption to tumor samples, we found that the performance of the ROC curve analysis of the collagen score for recurrence prediction was negligible, as illustrated in Figure 3G (AUC=0.48). Additionally, a high collagen score was not associated with recurrence (P=0.729) in our PCR cohort, in contrast to the 5-gene signature (P<0.0001). In GSE14333 and GSE33113, the collagen score was also not associated with recurrence (P=0.271 and P=0.369; there is an association for GSE17538). Regarding DFS time, the categorization of our cohort (PCR dataset) according to this collagen score (high and low categories, using the 3^rd^ tertile as a cut off) were not related to clinical outcome (log-rank test, P=0.938; Figure 3H; P=0.785 using the median collagen score as cut off) and the same results were obtained in GSE14333 (P=0.525, Supplementary figure 2D). Collagen score as a continuous variable was not associated with DFS (Cox regression analysis, P=0.904). Thus, many high collagen samples were assigned as low-risk (Figure 3I). We also used immunohistochemical staining to evaluate PDLIM3 in high-stroma tumors quantified in H-E slides as the tumor/stroma ratio. As shown in Figure 3J, two high-stroma tumors, one classified as high-risk according to the 5-gene signature score, with intense α-SMA and PDLIM3 staining, and the other classified as low-risk according to our gene classifier, with intense α-SMA but without PDLIM3 positivity. Additionally hematoxylin-eosin evaluation of stroma percentage in these samples reported no statistically significant differences between high stroma and low stroma groups (Kaplan-Meier Log Rank P = 0.123; Supplementary figure 3A). Since neither the quantity of collagen nor the stroma percentage provided consistent results regarding patient's outcome, we confirmed by means a Kaplan-Meier analysis that the 5-gene classifier can help to stratify highly desmoplastic tumours according to recurrence risk. Accordingly, using the median Collagen score value as a cut off, in high collagen score samples, the 5-gene classifier clearly stratify patients according to risk (HR = 14.47, P = 0.00036), while in low collagen score samples did not (HR = 1.56, P = 0.344). This association is also illustrated in supplementary figures 3D-to-F stratifying patients in three groups according to Collagen score. The same trend is observed in dataset GSE17538 (supplementary figures 3G-to-I).

Additionally, we also investigate this fact in tumors regarding the tumor/stroma percentage assessed by hematoxylin-eosin staining. The 5-gene classifier also stratifies patients according to risk of relapse in the high stroma group (Supplementary figure 3J and K).

### Performance of the 5-gene classifier excluding very low-stroma samples

We assumed, given the aforementioned results, that samples with a low collagen score might be misclassified due to the low level of CAFs in such specimens. To prove that, we excluded samples with collagen score < percentile 25. After discarding these samples, as proof of concept, the cohort comprised 107 patients, with same stages proportionality. As demonstrated by the Kaplan-Meier curves (Figure 3K and supplementary figure 3B and C), the prognostic power of the 5-gene classifier is considerably superior. Therefore, as shown in Table 2, the assessment of the 5-gene classifier in samples relatively enriched in the stromal compartment differentiated patients into two risk groups. Actually, as illustrated in supplementary table 2 (ROC analysis), the predictive capability of the 5-gene classifier increases as we discard samples according to their collagen score. Thus a cohort containing samples with low collagen scores has a smaller AUC than a cohort enriched in samples containing higher collagen scores. The predictive power increases if Collagen is present, assuming that Collagen means fibroblasts. To corroborate this fact, we performed an interaction analysis between the collagen score and the 5-gene classifier (Supplementary table 3) to demonstrate that the classifier has better predictive capabilities in samples with a higher collagen score.

Thus, selecting the 107 samples with high collagen scores (above the 25^th^ percentile), the 5-gene classifier is still the strongest predictor of DFS in univariate and multivariate analyses (univariate: HR=6.87, 95% CI=3.02-15.49, P<0.0001; multivariate: HR=6.18, 95% CI=2.61- 14.6, P<0.0001; Table 3). Using the 5-gene signature score as a continuous variable, the HR increased by 1.36 (95% CI=1.18-1.57, P<0.0001) per unit increase in the SD of expression. The 5-gene signature was also significant in multivariate analysis when used as a continuous variable (HR=1.34, 95% CI=1.14-1.58, P<0.0001). In addition, post-test probabilities (Table 2) demonstrated the suitability of the 5-gene classifier for clinical practice.

**Table 3:** Univariate and multivariate analysis for DFS in quantitative RT-PCR validation dataset

	univariate			multivariate		
	P value	HR	95,0% CI	P value	HR	95,0% CI
**Initial cohort (n=142)**						
Gender (female vs male)	0.07	0.55	0.29-1.04			
Age >55 years	0.88	0.93	0.37-2.36			
Stage (3 vs 2)	**0.013**	2.33	1.19-4.53	0.084	1.91	0.92-3.97
Location (rectum vs colon)	0.055	1.79	0.99-3.26			
Grade (high vs low)	**0.033**	2.42	1.07-5.45	0.52	1.33	0.56-3.16
isolated nodes (<14 vs >14)	0.063	1.77	0.97-3.24			
Lymphatic invasion (no)	**0.049**	0.53	0.28-0.99	0.37	0.73	0.37-1.45
Adjuvant chemotherapy (no)	0.083	1.71	0.93-3.14			
Collagen score (high vs low)	0.689	0.88	0.46-1.66			
5-gene classifier (high vs low)	**<0.0001**	3.14	1.72-5.72	**0.002**	2.67	1.42-5
5-gene classifier (continuous, +1SD)	**0.005**	1.19	1.05-1.34	0.032^*^	1.16	1.01-1.32
**>percentil25 Collagen score (n=107)**						
Gender (female vs male)	0.115	0.61	0.28-1.3			
Age >55 years	0.7	0.79	0.24-2.6			
Stage (3 vs 2)	**0.014**	2.76	1.23-6.21	0.363	1.57	0.67-3.69
Location (rectum vs colon)	**0.003**	2.99	1.44-6.23	0.009	2.76	1.3-5.9
Grade (high vs low)	**0.023**	2.82	1.15-6.93	0.78	1.14	0.45-2.94
isolated nodes (<14 vs >14)	0.067	1.95	0.95-4			
Lymphatic invasion (no)	0.052	0.48	0.23-1			
Adjuvant chemotherapy (no)	0.51	1.27	0.62-2.61			
Collagen score (high vs low)	0.361	0.71	0.34-1.47			
5-gene classifier (high vs low)	**<0.0001**	6.87	3.02-15.49	**<0.0001**	6.18	2.61-14.6
5-gene classifier (continuous, +1SD)	**<0.0001**	1.36	1.18-1.57	<0.0001^*^	1.34	1.14-1.58

* Multivariate analysis including Stage, location, grade and 5-gene signature score as a continuous variable.

## DISCUSSION

In this paper, we report the development, validation and technique translation (RT-PCR) of a 5-gene classifier (including risk genes *ULBP2, SLC7A2, PDLIM3, AMIGO2*, and the protective gene *CCL11*) derived from a 108-gene expression signature obtained from a microarray of deregulated genes in CAFs from colorectal cancer (Molecular Oncology 10.1016/j.molonc.2014.04.006). The 5-gene classifier is associated with the risk of relapse in patients with Stage II and Stage III CRC, regardless of the number of fibroblasts in the tumor specimen.

Not all the cells in a tumor are transcriptionally equivalent, and this is the case for CAFs. This is a consequence of the spatial location of each cell within the tumoral microecosystem, arising first from the particular relationships with other cells and other cell types, and second from the anatomical demarcation of a tumor and the mechanical and compressive forces exerted by the surrounding tissue (tensegrity) [23, 24]. Thus, recently, greater complexity has been revealed in the form of the considerable inter-tumoral heterogeneity of CAFs in the same organ as we and others have reported (Molecular Oncology 10.1016/j.molonc.2014.04.006) [25]. This suggests that the physiological status of CAFs may be more relevant in terms of the prognostic information provided than the number of fibroblasts itself. Therefore, a key concept in this study is that multiple, distinct biological responses are present to different extents within the CAFs of a given tumor, and the balance between these responses determines the good or bad prognostic value. Accordingly, our unsupervised analysis of the most variable genes in isolated CAFs clearly segregated two different patterns. The first comprises CAFs with the four overexpressed risk genes (*AMIGO2, PDLIM3, ULBP2* and *SLC7A2*) and the underexpressed protective gene (*CCL11*). This subset is characterized by the overexpression of genes involved in the processes of migration, wound healing, angiogenesis, TGFβ responsive genes and downregulation of inflammatory genes. The second pattern involves a cluster enriched in inflammatory genes and the underexpression of the four risk genes. This is consistent with a previously observed stromal signature in breast cancer [26] in which samples from the good outcome cluster overexpressed a distinct set of immune-related genes. These good outcome tumors also have higher levels of fibroblasts expressing *CXCL14*, a chemokine that stimulates natural killer cell migration. Thus, individuals with this gene expression pattern may benefit from treatments targeting tumor cells via the immune response. In contrast, individuals with a poor outcome have higher levels of expression of stromal genes involved in wound healing and angiogenesis. Several matrix metalloproteases are also strongly expressed.

The prognostic utility of stroma has recently been more widely reported although it is still not used routinely. Some authors have proposed using the percentage of tumor/stroma as a tool for classifying outcome in a variety of cancer types [27-30], even though these methods have some bias and do not take into account the intratumor heterogeneity with respect to transcriptomic status. In our cohort, the tumor stroma percentage did not associate with prognosis (Supplementary figure 3A), at least in the 106 cases available, although a trend is observed. Other authors evaluated the prognostic value of stroma or particular stromal cell types from the point of view of their transcriptional status [26, 31-33]. In relation to other CAF-specific genes our 5-gene classifier predicted recurrence with higher accuracy (Supplementary table 4). Interestingly, the 5-gene classifier is able to discriminate the *a priori* bad outcome highly desmoplastic tumors into two groups of risk.

More recently, many of the new molecular classifications have considered the stromal contribution to prognosis [34-36] [37] [38]. We only found overlap between two genes of our classifier and a recent molecular classification [38]. We were not surprised at this since the small number of genes in our classifier and its origin is based on the transcriptional changes between NCF and paired CAFs. By contrast, we found considerable overlap with the signature derive from other CAFs signatures from breast, lung, oral squamous cell carcinoma and esophagus [32, 39-41].

Integrating all the available information, it can be concluded that the stroma plays an extremely important role in the prognosis of colorectal cancer. Our results emphasize that the quantity of CAFs in a tumor is not as important as their transcriptomic or physiological status. Our 5-gene classifier, comprising genes almost exclusively expressed by CAFs, seems to reflect the physiological state of these myofibroblasts rather than just the number of fibroblasts in the tumor stroma. By contrast, the collagen score reflects the quantity of fibroblasts in a given sample and could be used to estimate the desmoplastic reaction. The lack of prognostic value of the collagen score with respect to the binary event (recurrence) and disease-free survival, leads us to suggest that the quantity of CAFs is not important for prognosis. With respect to the genes of the classifier, four are considered to be risk genes, since their level of expression is positively correlated with the risk score, and *CCL11* is considered to be a protective gene, its value being negatively correlated with the risk score. This is consistent with the levels of expression in NCFs and CAFs used to develop the differential signature (Molecular Oncology 10.1016/j.molonc.2014.04.006). This can be explained in two ways. One possibility is that fibroblasts in a tumor have heterogeneous transcriptomic profiles, although all of them are considered as CAFs, which, depending on their interaction with their surroundings (i.e., crosstalk with other cells), express these genes in a particular manner, leading to distinct CAF subpopulations coexisting in a tumor. The net balance of gene expression, rather than the quantity of fibroblasts, is what ultimately determines the prognostic value. In other words, the positional demarcation of a fibroblast in a tumor determines the crosstalk with its surroundings, and this can therefore be measured in terms of mRNA transcription. Another explanation for such CAF heterogeneity is that there are different origins [42].

Remarkably, the 5-gene classifier identifies Stage-II patients at high risk of relapse independently of the pT, and Stage-III patients at low risk of relapse. We found that the 5-year DFS rate was >90% in Stage-II patients who had a low 5-gene classifier score. This rate could be extended to Stage-III patients when selecting an appropriate biopsy from the surgical specimen to perform the test. This suggests that adjuvant chemotherapy would be of minimal benefit to this group of patients, especially those classified as Stage-II. Some authors have reported that the association of stromal gene expression with colorectal recurrence may explain the AJCC staging in which invasion is the critical feature [43]. Our study suggests that there is no statistical association with disease stage. Multivariate Cox regression analysis confirmed that the classifier, either as a dichotomized or a continuous variable, is an independent prognostic factor in Stage II/III colorectal cancer. Additionally, the way in which the classifier has been developed enhances the biological relevance of it component genes, since no regression coefficients were used to obtain the score, and the genes were weighted equally.

The performance of the 5-gene classifier in independent, whole tumor-derived data sets and with two distinct technologies (microarray and PCR) indicates that although the prognostic power of the classifier is specific to tumor stroma, the signal can be detected when a minimal amount of stromal tissue is present in the sample. Moreover, prediction accuracy increases along with the quantity of stromal tissue, although this is not associated with a higher risk of relapse, as indicated by the lack of prognostic value of the collagen score.

The use and benefit of adjuvant chemotherapy in Stage-II patients is controversial [4], but the QUASAR study suggested that a subset of Stage-II patients with higher relapse risk may benefit from adjuvant treatment. Stage-III patients are routinely offered adjuvant chemotherapy but, despite treatment, approximately 40% of them relapse [3]. Our 5-gene stromal classifier identifies subgroups with different recurrence risk even though there is a wider clinical margin between high-risk and low-risk patients in Stage-II compared with Stage-III patients.

## CONCLUSIONS

In conclusion, using the 5-gene classifier in a clinical setting can provide more accurate information about the risk of recurrence than is possible from conventional clinicopathological criteria alone, and may facilitate the selection of high-risk Stage-II patients who would benefit from adjuvant therapy. Additionally, the predictive power of the 5-gene classifier is precise and accurate when selecting an area/biopsy from the surgical specimen that is relatively rich in tumor stroma (Figure 3K), as previously reported in prostate cancer [44]. The small number of genes comprising this classifier, and its validation in RT-PCR make it more amenable to clinical use than are large signatures. As is the case for other studies that have developed signatures, a limitation of this one is that it has only been possible to carry out a retrospective analysis of prospectively collected specimens. Future prospective studies are needed to confirm whether Stage-II patients at high-risk of relapse based on our classifier can achieve a better outcome if they receive adjuvant chemotherapy. Nonetheless the extensive *in silico* validation, and the translation and validation with PCR demonstrate the robustness of this 5-gene stromal classifier.

## METHODS

### Training and validation series: patients

All public datasets were obtained from the GEO (Gene Expression Omnibus) and corresponded to the gene expression profile (GEP) of CRC patients obtained with the microarray platform (Affymetrix Human Genome U133Plus 2.0). GSE14333 was used as the training set. Datasets used for external validation corresponded to the GEP of whole tumor samples and GSE33113, GSE17538, and GSE14095. GSE31595 and GSE26892 were included in the meta-analysis. Datasets GSE18105 and GSE21510, containing GEP of LCM (laser capture microdissection) of epithelial cells from CRC patients, were used for negative validation of the stromal classifier. Table 1 illustrates the characteristics of the patients in the training set, the *in silico* validation metacohort and the RT-PCR validation cohort.

### Specimen characteristics

Training and validation datasets obtained from GEO corresponded to whole tumor specimens. PCR validation dataset consisted in retrospective 142 frozen whole tumor samples stored at −80ºC and collected from the Hospital Universitari de Bellvitge Tumor Bank from 1996 until 2001.

### Classifier development

Figure 1 depicts the process by which the classifier was developed. The 5-gene classifier is derived from a 108-gene signature of differentially expressed genes (DEGs) between carcinoma-associated fibroblasts (CAFs) and paired normal colonic fibroblasts (NCFs) (Molecular Oncology 10.1016/j.molonc.2014.04.006).

### *In silico* validation

The optimal predictive classifier was validated in three independent datasets (GSE17538, GSE14095 and GSE33113). GSE14333 and GSE17538 cohorts were partially overlapping. Cases duplicated in GSE14333 and GSE17538 were excluded from validation. We also performed a meta-analysis, pooling GSE26892 and GSE31595 with the aforementioned GSE17538 and GSE33113 datasets (metacohort n=317; Table 1). In order to remove systematic biases between datasets, the expression levels of all genes were transformed to z-scores before pooling. A recurrence score was computed for each sample (see the Supplementary Methods).

### Gene expression analysis by qRT-PCR in validation-PCR dataset

Frozen primary tumor tissue samples (n=142) from colorectal cancer patients (62 Stage-II, 80 Stage-III) were collected from the Hospital Universitari de Bellvitge Tumor Bank. Clinical and pathological data of these patients were obtained from the medicals records and reviewed for the study. RNA isolation and real-time PCR procedures are detailed in the Supplementary Methods.

### RNA isolation and real time PCR procedures

RNA was extracted from whole tumor tissue (both stroma and epithelial compartment) using TRIzol^®^ reagent method and column purification using PureLink ™ RNA Mini Kit (Invitrogen). RNA quantity was determined by NanoDrop ND-1000 spectrophotometer (NanoDrop Technologies Inc, Rockland, DE) and 100ng of total RNA was reverse-transcribed using M-MLV Reverse Transcriptase (Invitrogen) following manufacturer's instructions. A 0.1μg equivalent of the corresponding cDNA was used for each quantitative PCR assay performed with the LightCycler^®^ 480, SYBR Green I Master (Roche Applied Science, Mannheim, Germany). Primers were designed using Primer3 Input (http://primer3.wi.mit.edu) and predicted PCR product sequences were verified by using BLAST (http://www.ncbi.nlm.nih.gov/blast). All primer sequences will be provided upon request. In every qRT-PCR reaction, a standard curve made from serial dilutions of a mix of RNA from different isolated fibroblasts was added to extrapolate for cDNA amount of the specific gene. To control for variation in RNA quantity between samples, the expression of each gene was further normalized to the geometric mean of two specifically selected reference genes. We aimed to use a reference gene in common at least between NCF, CAF and colorectal cell lines, attempting to prevent that the ratio tumor/stroma affected the normalization of the classifier genes. Different possible housekeeping genes (*ACTB, PMM1, GAPDH, B2M, HPRT1, PPIA*, *IPO8, RSP13*) were tested for stability in 10 colon cancer tissue samples as well as in different colorectal cancer cell lines and in a mix of RNA from NCF and CAF and in mixes of NCF, CAF and CRC cell lines. Using RefFinder, a web-base tool (http://leonxie.com/referencegene.php) which integrates different online applications (*Normfinder [45]*, geNorm [46], *BestKeeper [47]* and *ΔCt [48]*), candidate reference genes mentioned above, were compared and ranked to select those with more stability. *ACTB* and *PMM1* were the most stable genes between all different samples tested. Normalized values were further log transformed and standardized.

### Recurrence score estimation

A recurrence score for a given patient was calculated as the sum of the standardized value of each gene. No gene level parameter estimation was performed and coefficients were established as 1 for overexpressed genes in CAF vs. NCF (*PDLIM3, SLC7A2, ULBP2* and *AMIGO2*; risk genes) and -1 for underexpressed gene in CAF vs. NCF (*CCL11*, protective gene). Patients in each cohort were classified according to their 5-gene expression score as having a high-risk gene signature or a low-risk gene signature. As a cut off we used the 3^rd^ tertile of the classifier score (percentile 66.66) in the training set. Then low-risk patients were define as those with score < 1.1328 and high-risk patients as those above 1.1328. The same cut off value was applied to all other validation cohorts

### Statistical analysis

Kaplan-Meier survival analyses with the log-rank test were used to estimate five-year disease-free survival (DFS) and disease-specific survival (DSS) rates in the different cohorts tested. DFS and DSS were defined as the time until an event (relapse, locoregional or metastasis, and death from CRC, respectively), and patients were censored at last follow-up, cancer-related death or treatment-related death. In all datasets, a minimum three-year follow-up was required for patients without tumor recurrence. More detailed information is provided in supplementary statistical methods.

## SUPPLEMENTARY METHODS FIGURES AND TABLES




