# Supplementary material for: A 5-gene classifier from the carcinoma-associated fibroblast transcriptomic profile and clinical outcome in colorectal cancer
Source: Oncotarget. 2014 Jul 23;5(15):6437–52. doi: 10.18632/oncotarget.2237 (PMC4171642; doi:10.18632/oncotarget.2237)
Supplement: Supplementary file 1 [file oncotarget-05-6437-s001.pdf]

SUPPLEMENTARY METHODS.

Classifier stromal specificity.

To confirm stromal specificity of the classifier identified, we tested its ability in predicting recurrence in GSE18105 and GSE21510 (LCM tumour samples enriched in epithelial cells). In addition, CAF-specificity expression of the signature genes was checked in GSE39396 (cell-type specific expression profile of CRC population from six patients). We previously validated cell specificity using different NCF, CAF and CRC cell lines (Molecular Oncology 10.1016/j.molonc.2014.04.006).

Supplementary statistical analysis.

The potential usefulness of the classifier in clinical practice was evaluated by means of positive and negative likelihood ratios (LR+ and LR−, respectively) and predicted positive/negative post-test recurrence probabilities. In the qRT-PCR data cohort, to determine the independence of the CAF-derived signature to clinicopathological variables in predicting an individual's risk of relapse and cancer-specific survival, we performed a Cox proportional-hazard multivariate analysis, including the variables that were significant in the univariate analysis. This analysis could not be done using public datasets because few clinical data were available. All analyses were done with SPSS version 21.0.

Epithelial-to-menchymal transition (EMT)

We explored a possible contribution of epithelial cells in EMT accounting for the expression of the five genes of the classifier, since the detected expression in tumours could have been masked by cells in EMT in those tumours and not exclusively expressed by CAFs. To discount this possibility, we cultured two cells lines (DLD1 and SW620) with one of the most efficient EMT inducers (HGF) during 8 hours. We assessed the levels of EMT associated genes plus the 5 genes of the classifier by means of real time PCR.

Additionally we cultured DLD1 cells in conditioned medium from CAFs for 24h, as we previously described the induction of EMT by factors released by fibroblasts. We compared expression values with those from CAFs (assessed by RT-PCR).

CAF heterogeneity.

Robust multi-array average values of thirteen samples of CAFs were used to select probes with the highest variability (standard deviation). Probes with RMA values > 4 and standard deviation > 0.75 were chosen. Among all most variable probes, those with a known gene associated were selected for further analysis (880 genes). Unsupervised clustering analysis was performed for grouping fibroblasts samples based on the expression of those genes.

Additionally, we cross-referenced genes differentially expressed in each CAF subtype against gene ontology annotations (DAVID database) to identify overrepresented gene ontology biological processes using a significance threshold of FDR q value < 0.1.

Tumour-stroma ratio assessment.

The tumor-stroma ratio was determined by two observers (XS, MJP) on hematoxylin and eosin staining in all the slides available for 106 out of 142 cases. Visual estimation of the percentage of stroma cells in was determined using 10X magnification lens. Pathologists scored the percentage in multiples of 5. Low stroma samples were defined as having 50% or less stroma area. Consequently, high stroma samples were defined as having 55% or more stroma ratio. In the case of tumour heterogeneity concerning the stromal presence, the area with the highest amount of stroma was used to determine the final result. Only areas that were surrounded by tumour cells in all directions were considered, so as to avoid scoring peripheral regions at the edge of the tumour.

SUPPLEMENTARY RESULTS

Using GSE39396, corresponding to four sorted cell populations (endothelial cells, inflammatory cells, epithelial cells and carcinoma-associated fibroblasts) from six colorectal tumours, we checked the main source of expression for each of the five genes. As depicted in Supplementary Figure 1A, *PDLIM3, CCL11, AMIGO2* and *SLC7A2* are basically expressed only by fibroblasts. The fifth gene, *ULBP2* is consistently expressed in endothelial cells and epithelial cells. Additionally, to ascertain that the expression of these genes belongs to CAFs instead of being epithelial cells we checked the performance of the classifier in epithelial cell enriched samples (laser capture microdissection). As depicted in supplementary figure 1B and C, the 5-gene classifier did not have predictive power over recurrence in these samples. Additionally, we checked a possible contribution of epithelial cells in EMT (DLD1 and SW620) accounting for the expression of the classifier genes, but after stimulation with HGF, the genes of the classifier did not markedly increase (Supplementary Figure 1F), even in cells undergoing an EMT process (according to *SLUG* and *ZEB1* mRNA expression levels; Supplementary Figure 1F, right panel). To rule out the possibility that epithelial cells in EMT instead of fibroblasts are responsible for the

values detected in tumour specimens, we also evaluated the levels of classifier genes in tumour cell lines cultured with conditioned medium from CAFs. Except for ULBP2, the expression values were far from similar to those of CAFs (*in vitro*; Supplementary Figure 1G). Considering all these results, we concluded that the epithelial cells in EMT did not mask the performance of the classifier.

Supplementary Table S1. Association of clinicopathologic variables with 5-gene signature score (high-risk or low-risk) in the Q-PCR validation dataset (n=142).

	Low risk	High risk	P value
**Group size**	98	44	
**Gender**			0.123
Male	51 (51.5)	29 (67.4)	
Female	47 (48)	15 (34.1)	
**Mean age**	66.8	66.4	0.8
>55 years	85 (86.7)	40 (90.9)	0.48
<55 years	13 (13.3)	4 (9.1)	
**Stage**			0.123
Stage II	47 (48)	15 (34.1)	
Stage III	51 (52)	29 (65.9)	
**Location**			0.406
Colon	65 (66.3)	26 (59.1)	
Rectum	33 (33.7)	18 (40.9)	
**Grade**			0.001
Low	94 (95.9)	34 (77.3)	
High	4 (4.1)	10 (22.7)	
**Isolated nodules**			0.287
>14	69 (70.4)	27 (61.4)	
<14	29 (29.6)	17 (38.6)	
**Adjuvant chemotherapy**			0.452
yes	49 (50)	25 (56.8)	
no	49 (50)	19 (43.2)	
**Lymphatic invasion**			0.217
yes	22 (22.7)	14 (32.6)	
no	75 (77.3)	29 (67.4)	
**KRAS status**			
wt	54 (55.1)	19 (43.2)	0.485
mut	32 (32.6)	15 (34)	
NA	12 (12.3)	10 (22.8)	
**MSI-high**			0.429
yes	15 (15.3)	4 (9)	
no	70 (71.4)	30 (68.2)	
NA	13 (13.3)	10 (22.8)	
**Collagen score**			<0.0001
High	23 (23.5)	24 (51.1)	
Low	75 (76.5)	20 (45.5)	

	Low risk	High risk	P value
**Recurrence**			
yes	20 (20.4)	23 (52.3)	<0.0001
no	78 (79.6)	21 (47.7)	
**Mean time to relapse (years)**	7.23	5.14	0.003
**Mean survival time (years)**	7.76	6.12	0.009

Supplementary Table S2. ROC analysis.

	AUC 5-gene classifier (recurrence)	
	Full cohort (n=142)	0.64
Selecting samples Collagen score above:	>Percentile 10 (n=128)	0.72
	>Percentile 20 (n=114)	0.74
	>Percentile 30 (n=100)	0.75
	>Percentile 40 (n=85)	0.76
	>Percentile 50 (n=71)	0.81

The Area Under the Curve indicates the accuracy of the 5-gene signature for recurrence prediction. The AUC increases as we discard patients according to its Collagen score.

Supplementary Table S3. Interaction models for the 5-gene classifier and the collagen score in four cohorts used for event prediction as a function of time (Cox) and prediction of the binary response (recurrence).

		Cox regression (DFS) P-value	Log binary regression (recurrence) P-value
RT-PCR cohort	5-gene classifier<br>Collagen score<br>5-gene*collagen	0.023<br>0.029<br>0.0002	0.016<br>0.052<br>0.002
	5-gene classifier	0.005	0.009
	Collagen score	0.904	0.906
GSE17538	5-gene classifier<br>Collagen score<br>5-gene*collagen	0.00016<br>0.16<br>0.019	0.001<br>0.16<br>0.07
	5-gene classifier	<0.0000001	0.000006
	Collagen score	0.0005	0.0003
GSE33113	5-gene classifier<br>Collagen score<br>5-gene*collagen	0.035<br>0.195<br>0.179	0.045<br>0.2<br>0.187
	5-gene classifier	0.016	0.025
	Collagen score	0.07	0.08
GSE14333	5-gene classifier<br>Collagen score<br>5-gene*collagen	<0.0000001<br>0.281<br>0.6	0.00002<br>0.43<br>0.76
	5-gene classifier	0.000001	0.000005
	Collagen score	0.314	0.15

In grey shaded rows we illustrate the interaction models and in white rows the principal components as univariate analysis to remember the significance in univariate analysis.
When Pvalue is <0.05 for the product of the two variables the interaction is positive.

Supplementary Table S4. Comparison between different previously reported genetic classifiers in the dataset GSE17538 and GSE33113 used in the study.

	GSE17538		GSE33113	
	**HR**	**P**	**HR**	**P**
**5-gene classifier**	1.38	0.000019	1.19	0.052
Oncotype (no gene weight)	0.98	0.8	1.02	0.82
**5-gene classifier**	1.33	0.00047	1.1	0.34
Oncotype	1.1	0.58	1.65	0.052
**5-gene classifier**	1.36	0.000008	1.2	0.02
Coloprint	1	0.851	1.01	0.78
**5-gene classifier**	1.36	0.00019	1.11	0.301
CAFs specific genes	1	0.924	1.13	0.09
**5-gene classifier**	1.34	0.002	1.13	0.241
CAFs classic genes	1.03	0.761	1.14	0.278

We compared the performance of the genes of our classifier in relation to previous reported classifiers and two different CAF-specific signatures. GSE39396 consist in FACS sorted cells from six colorectal tumours (EPCAM+, FAP+, CD31+ and CD45+). We used the best CAF-specific genes according to FAP+ cells from dataset GSE39396 (*COL1A1, COL3A1, LUM, DCN* and *CTSK*, combined score), although these five genes might be just remarking the mesenchymal origin of these FAP+ cells. For that reason we also checked the performance of classic genes associated with CAFs like *FAP*, endosialin *CD248* and alpha smooth muscle actin (combined score).
We just applied the gene weight parameter in the case of comparison with Oncotype (as reported in the website www .oncotypedx.com; −0.30 for cell cycle genes *MYC*, *Ki67* and *MYBL2*, 0.15 for stroma genes *FAP*, *BGN* and *INHBA* and 0.15 for *GADD45B*). In the case of Coloprint, no gene weight were applied since these values have not been reported so far. To avoid collinearity, we compared tests two-by-two using a multivariate Cox regression for DFS.

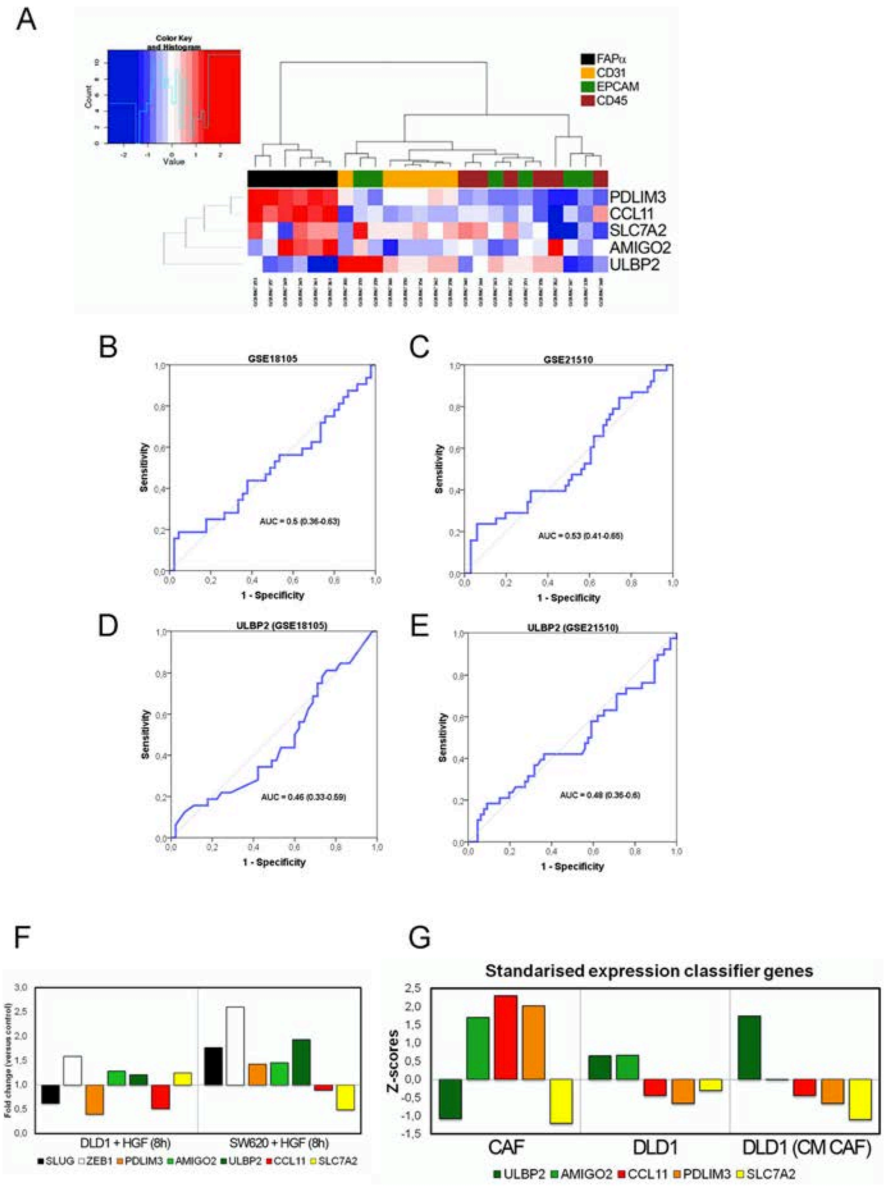

Supplementary Figure S1: (A) Unsupervised heatmap of the genes of the classifier with respect to their expression in the GSE39396 dataset. The expression of the five genes was checked in 4 CRC cell populations (obtained from six colorectal cancer patients): FAPα + (fibroblasts), EPCAM+ (epithelial cells), CD45+ (leukocytes) and CD31+ (endothelial cells). Gene expression levels enabled CAFs to be clearly differentiated.

(B and C) Receiver operating characteristic curves showing the performance of the 5-gene classifier for predicting recurrence in epithelial cell enriched datasets. Both datasets GSE18105 (B) and GSE21510 (C) displayed the curve (blue line) over the dotted grey line, indicating random prediction in these two datasets. This is due to the stromal specificity of the genes of the classifier.

(D and E) since *ULBP2* was the only gene that its expression can be also detected in endothelial cells and epithelial cells (as illustrated above in the heatmap), we checked the predictive capabilities of this gene in epithelial cell enriched samples. As displayed in both datasets of laser capture microdissected samples, epithelial expression of ULBP2 has no predictive power over recurrence in epithelial cells. Therefore, probably the *ULBP2* expression in the stroma is contributing to the good performance of the 5-gene classifier rather than the expression in the epithelial compartment.

(F) The expression level of the five genes of the classifier detected in tumours could have been masked by cells in EMT in those tumours and not exclusively expressed by CAFs. To discount this possibility, we cultured two cells lines (DLD1 and SW620) with one of the most efficient EMT inducers (HGF). As depicted in the coloured bars, after 8 h of treatment with HGF, mRNA values of the genes of the classifier remained roughly constant, even in cells probably undergoing an EMT process (SW620), since values of *SLUG* and *ZEB1* were considerably higher than those of controls without HGF.

(G) To further rule out any possibility of the expression values of tumour cells in EMT helping to mask the fibroblast specificity of the genes of the classifier, we cultured DLD1 cells in conditioned medium from CAFs. Except for *ULBP2*, the value of the other four genes was much lower than those of the CAFs (assessed by RT-PCR).

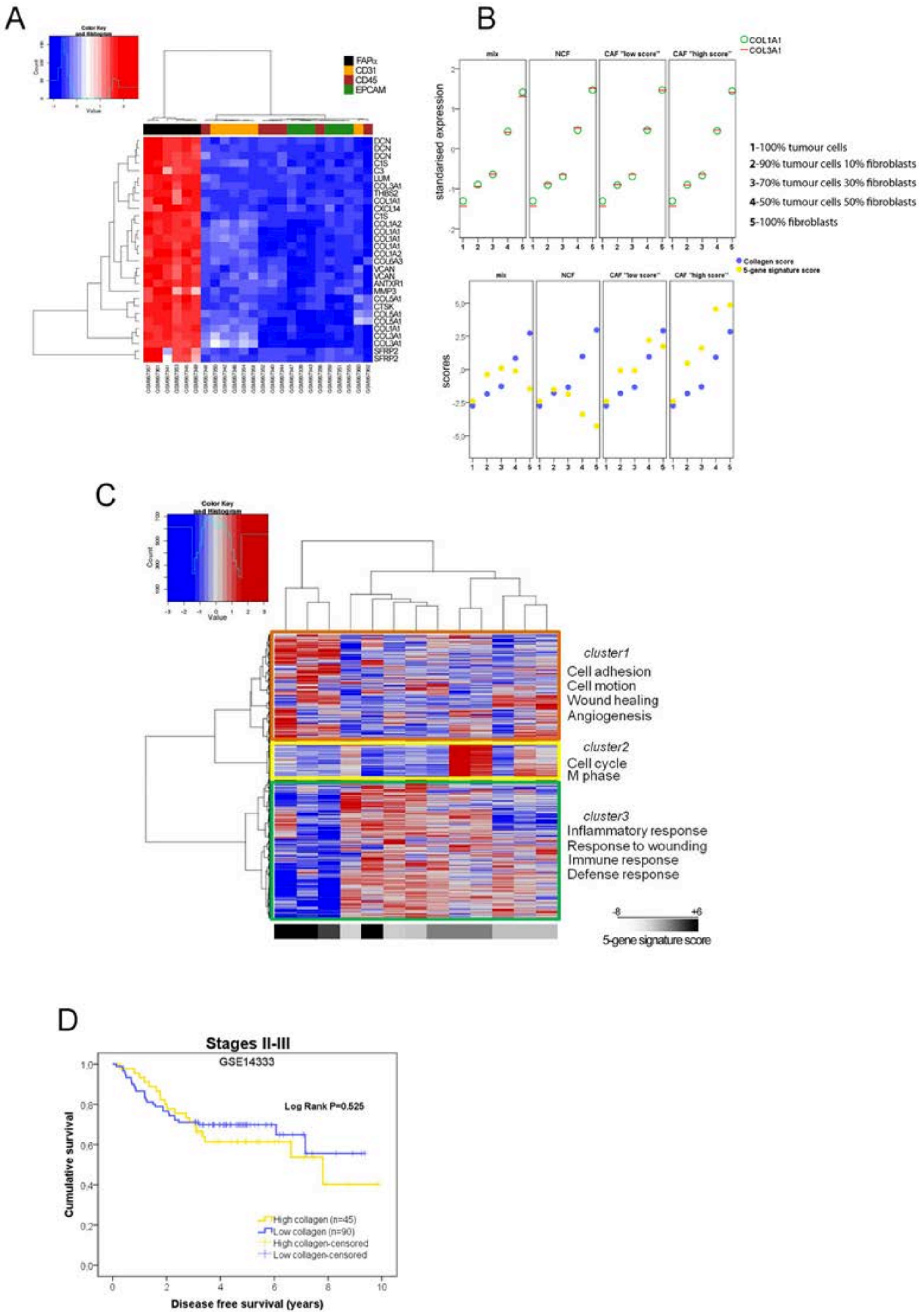

Supplementary Figure S2: (A) Unsupervised heatmap of the dataset GSE39396 to select those genes most specifically associated with fibroblasts (and less variable among this cell type) in comparison with other cell types isolated from six colorectal tumours (fibroblasts depicted in black, endothelial cells in yellow, inflammatory cells in brown, and epithelial cells in green). We selected *COL1A1* and *COL3A1*, these being the genes with the lowest variance in fibroblasts, to calculate a collagen score.

(B) We created artificially cell mixtures with different proportions of tumour cells (a pool of five colorectal cancer cell lines) and fibroblasts with different 5-gene classifier scores (NCF, with a very low score compared with the other two CAFs used, a CAF "low score" and a CAF "high score"). With these cells, we prepared four sample types: mixtures of tumour cells and the same proportion (1/3) of

each of the fibroblasts; mixtures of tumour cells with NCF; mixtures of tumour cells and "low score" CAFs; and tumour cells with "high score" CAFs. We prepared samples with a range of different ratios of epithelial and mesenchymal cells, from 100% tumour cells to 100% fibroblasts. The upper panels illustrate the standardised concentration of the two collagens used to calculate the collagen score (z-score averages for COL1A1 and COL3A1). These two genes were selected because they were the least variable and most specifically expressed genes in fibroblasts, based on data from GSE39396. For the four cell mixtures, as the percentage of fibroblasts increases, the expression values of the two genes also increase, showing that the more fibroblasts a sample has the more collagen it expresses. On the other hand, when we evaluated the 5 genes of the classifier in the same cell mixtures, depending on the fibroblasts' transcriptomic status, there was a correlation between the number of fibroblasts and expression of the genes of the classifier. Thus, our 5-gene classifier, comprising genes almost exclusively expressed by CAFs, seems to reflect the physiological state of these myofibroblasts rather than just the number of fibroblasts in the tumour stroma.

(C) CAF clustering and functional enrichment analysis of the most variable and differentially expressed genes.

Considering that the 5-gene classifier reflects the physiological state of the CAFs rather than the number of fibroblasts in the tumour, we concluded that different CAF phenotypes might be present as a consequence of different heterotypic interactions, thereby representing a source of intratumoral heterogeneity. To explore CAF heterogeneity we selected the genes with the greatest variability among 13 CAFs. Using a standard deviation cut-off value of > 0.75, which included 879 probes corresponding to 873 genes, we classified the CAFs by unsupervised clustering analysis. The CAFs segregated into two main clusters and the genes into three clusters. Gene ontology (http://david.abcc.ncifcrf.gov/) analysis of gene clusters reveals that cluster 1 is correlated with genes involved in migration, wound healing and angiogenesis. Interestingly, the four risk genes of the classifier (*AMIGO2*, *PDLIM3*, *ULBP2* and *SLC7A2*) occur in this cluster, along with other genes associated with tumour aggressiveness, like *IL6*, *LIF*, *SERPINE1*, *HBEGF*, *FAP*, *CCL7*, *CCL8*, *TGFB2*, *MMP1*, *MMP3*, *MMP12*, *POSTN*, *STC*1 and *TNC*, among others. Cluster 2 is correlated with cell cycle and mitosis genes and Cluster 3 aggregate genes of inflammatory response like *CCL11* (protective gene of the classifier), *CCL13*, *CXCL6*, *CXCL2*, *CXCL1*, *CXCL14*, *A2M*, *FOS*, *TLR3*, *IL6R*, *KIT* and *IL8* among others.

(D) According to the absence of prognostic value of the Collagen score, the same result was obtained from the GSE14333 dataset. The collagen score does not provide prognostic information. Higher collagen values are not associated with a worse outcome.

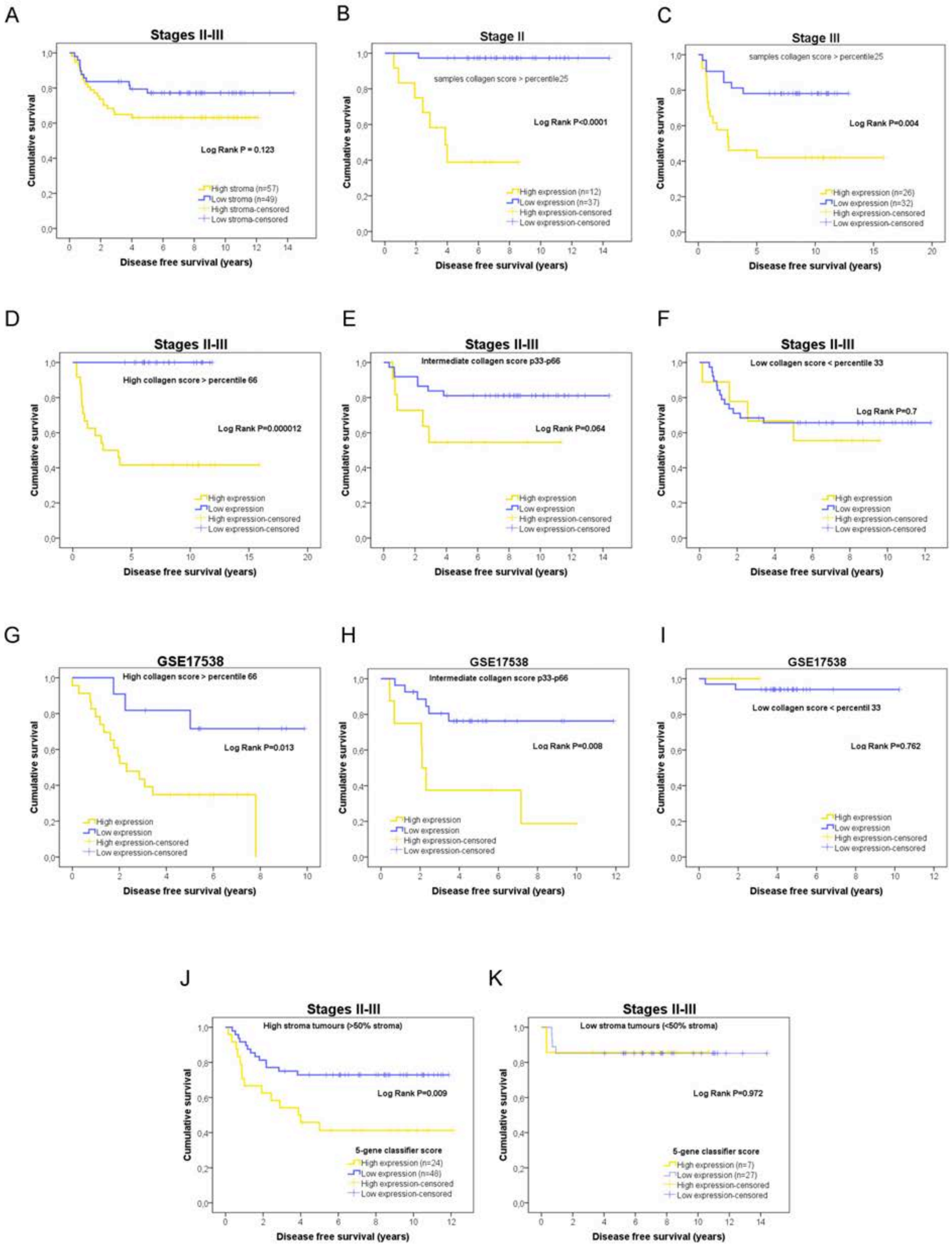

Supplementary Figure S3: (A) in 106 cases out of 142, an evaluation of the tumour/stroma ratio was scored by means of hematoxylin-eosin staining. Two pathologists recorded stroma percentage in multiples of 5 (5, 10, 15, etc). We used the mean of the two observations and the cut off of 50% to discriminate low stroma samples (10-50%) and high stroma samples (55%-90%). It is also

interesting to remark, that desmoplasia includes areas with high amounts of extracellular matrix components and cell types other than fibroblasts. For that reason the association between stroma ratio and CAFs quantity should be considered with caution.

(B and C) Kaplan-Meier survival plots after excluding samples below the 25th percentile of the collagen score (n = 107 patients), stratifying by stages.

Performance of the 5-gene classifier in patients according to their Collagen score: using tertiles of the collagen score, the 5-gene classifier clearly stratify risk of shorter DFS in those patients with high collagen score, illustrating the relevance of the transcriptomic status of CAFs for conferring tumor advantages **(D)**. Same trend is observed for intermediate collagen score samples **(E)** and no association is observed for low collagen score samples **(F)**, revealing probably a low quantity of mRNA transcripts for detection. Same observation was evidenced in GSE17538 **(G, H and I)**. To corroborate this results, when we applied the 5-gene classifier to high stroma samples, the classifier also stratified the patients according to two different groups of risk **(J)**. Again, no association was observed for low stroma samples **(K)**.
